# Radio Frequency Fingerprint-Based Intelligent Mobile Edge Computing for Internet of Things Authentication †

**DOI:** 10.3390/s19163610

**Published:** 2019-08-19

**Authors:** Songlin Chen, Hong Wen, Jinsong Wu, Aidong Xu, Yixin Jiang, Huanhuan Song, Yi Chen

**Affiliations:** 1 National Key Laboratory of Science and Technology on Communications, University of Electronic Science and Technology of China, Chengdu 611731, China; 2 School of Aeronautics and Astronautics, University of Electronic Science and Technology of China, Chengdu 611731, China; 3 School of Artificial Intelligence, Guilin University of Electronic Technology, Guilin 541004, China; 4 Department of Electrical Engineering, Universidad de Chile, Av Tupper 2007, Santiago 8370451, Chile; 5 EPRI, China Southern Power Grid Co. Ltd., Guangzhou 510080, China

**Keywords:** mobile edge computing, IoT, RF Fingerprinting, authentication

## Abstract

In this paper, a light-weight radio frequency fingerprinting identification (RFFID) scheme that combines with a two-layer model is proposed to realize authentications for a large number of resource-constrained terminals under the mobile edge computing (MEC) scenario without relying on encryption-based methods. In the first layer, signal collection, extraction of RF fingerprint features, dynamic feature database storage, and access authentication decision are carried out by the MEC devices. In the second layer, learning features, generating decision models, and implementing machine learning algorithms for recognition are performed by the remote cloud. By this means, the authentication rate can be improved by taking advantage of the machine-learning training methods and computing resource support of the cloud. Extensive simulations are performed under the IoT application scenario. The results show that the novel method can achieve higher recognition rate than that of traditional RFFID method by using wavelet feature effectively, which demonstrates the efficiency of our proposed method.

## 1. Introduction

In recent years we have seen an innovative Internet-of-Things (IoT) paradigm, which combines mobile edge computing (MEC) with traditional IoT architecture [1,2,3]. MEC is used as a bridge between IoT devices and remote cloud devices to provide edge intelligent services to meet the critical needs of industry digitization in terms of agile connectivity, real-time services, data optimization, application intelligence, security and privacy protection, which are key issues for the industry control applications [4,5,6]. However, such new architecture has aroused many security protection requirements, including security access authentication, security transmission, and data privacy etc., in which the most important one is security access authentication [7]. Due to constraints of terminals under the IoT system, and resource constraints of the existing authentication methods that rely on encryption, some lightweight and effective security access authentication measurements [8,9] are necessary. Recently, many researchers have turned to using the physical (PHY) layer information to enhance wireless security [10,11,12]. MEC operates on the wireless media. The innovative PHY-layer security designs can cope with the unique PHY layer weakness of the MEC in which physical characteristics, such as the channel responses between communication peers, the hardware property of the wireless transmitter, have been explored as a form of fingerprint in the scenario of wireless security.

Many scholars have made contributions to the development of physical layer security [13,14,15]. RFFID is of vital importance to physical layer security technology. In fact, radiofrequency fingerprints (RFF), which embody the hardware property of the wireless transmitter to be identified and have the characteristics difficult to be cloned, are a good candidate to be used to enhance device identification [16,17,18,19,20,21]. Additionally, RFFID is a lightweight authentication method for the transmitters, because the authentication algorithm is mainly performed on receivers and transmitters that do almost nothing. Therefore, it is especially suitable for the source-constrained terminals of IoT to perform access identification.

The RFFID method is different from the device signal authentication method proposed in [22]. The stochastic features of dynamic watermarking signal are used as identity information. Hall et al. [23] first proposed RF fingerprinting technology in Bluetooth wireless network device identification research in 2003. After that, studies have found that the transmitter can also be identified by transmitting the steady-state portion of the signal. Hu et al. [24] utilized RF signals to identify mobile phones in a mobile cellular network. Hall et al. [23] and Ureten et al. [25,26] used RF fingerprinting technology to achieve wireless positioning and access control for wireless network. In order to further improve the authentication rate of RFFID, a machine learning algorithm has been introduced in extensive research as the classification algorithm of RFFID [27,28,29]. However, a machine learning algorithm needs a certain amount of computing resources to ensure a higher recognition and authentication rate. Especially in offline training, the number of offline training samples will affect the effect of machine learning. With available computing resources, MEC can perform limited tasks in offline training of machine learning. When a large number of samples need to be trained, while uploading these computing cost tasks to cloud computing platform, the authentication rate can be expected to be further improved. In this article, we propose an efficient and flexible RFFID-MEC authentication method, in which RFFID is combined with MEC and the cloud, making full use of the characteristics of MEC-IoT framework to establish the two-layer model. The first layer provides data collection, extraction of RF fingerprint features, dynamic data storage and access authentication decision, which consumes less limited computing resources running at the MEC platform. The second-layer provides powerful computing for more complex and resource-consuming tasks in the remote cloud, such as feature learning, generating decision model, and establishing a machine learning algorithm. Since the authentication algorithm is mainly performed on the MEC, the terminals do almost nothing. The novel model, the edge computing, and cloud computing work collaboratively to ensure that the method has strong computing resources and improve the authentication rate. Compared with the conventional physical authentication method [10,11,12,30,31,32,33], our method makes efficient use of the characteristics of edge computing to collect transmitting signals and computing support of the cloud, and performs the fast identity authentication of terminals in the IoT scenario with asymmetric computing resources. Therefore, our proposed novel authentication scheme is light-weight to the IoT terminals. Our contributions can be summarized as follows:

(1) To the best of our knowledge, we are the first to propose the radio frequency fingerprint-based authentication, that combines physical characteristics of wireless device radio frequency and machine learning algorithms under the collaborative work of edge computing and cloud computing to achieve fast and efficient authentication.
(2) We present the typical scenario that uses an RFFID-MEC method for IoT devices authentication applications and demonstrate the effectiveness of the algorithm.

The rest of this article is organized as follows. In Section 2, we introduce the related work about the background information of MEC-IoT architecture and RFFID. The secure access authentication method based on RFFID- MEC is proposed in Section 3. Section 4 includes the application of the novel method to typical scenario and the evaluation of the proposed methods via experiments. Finally, the conclusions are given in Section 5.

## 2. Background of RFFID-MEC

### 2.1. MEC Architecture in IoT

**Figure 1:**
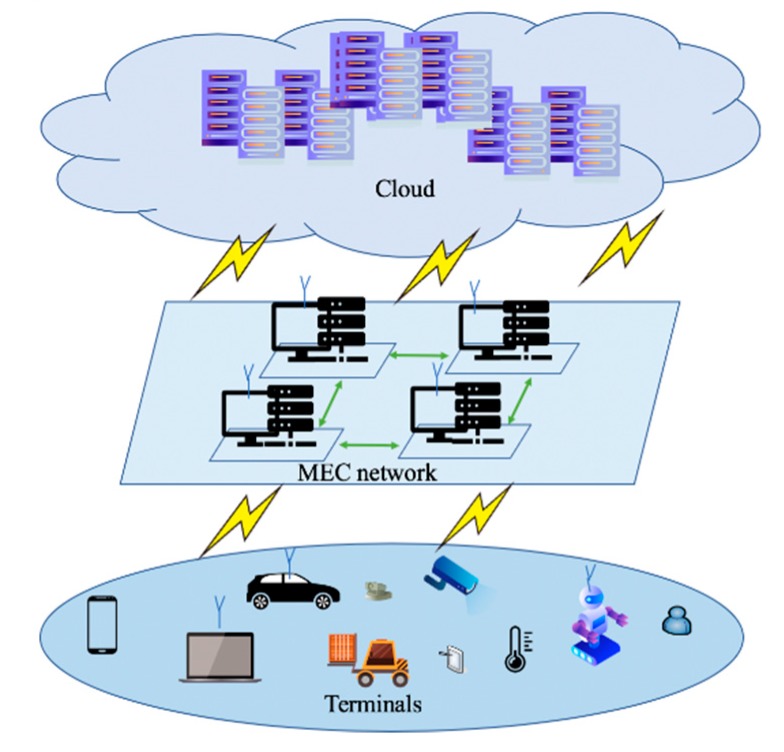
Mobile edge computing-Internet-of-Things (MEC-IoT) architecture.

The emergence of MEC brings a high-performance computing platform that provides data preprocessing, storage, and edge intelligent services [2]. As shown in Figure 1, the MEC-IoT architecture encompasses three different layers, the IoT devices, MEC, and the remote cloud platform. Each layer is characterized by different constraints on computation ability, memory, and energy availability. Among them, the computation ability of IoT terminals is the weakest. The MEC layer can perform limited tasks with available computing resources and the cloud layer provides strong computing support to the other two layers.

A key transformation is to perform information processing based on servers at network edge, applying the concepts of cloud computing. MEC can be seen as a cloud server running at the edge of a mobile network and performing specific tasks that are necessary to some scenarios, such as agile connectivity, real-time services, security and privacy protection, which could not be achieved with traditional IoT network infrastructure [1]. In addition, IoT devices are connected with MEC that provide (when needed) the computational resources for more complex and resource-demanding application or processing tasks. MEC devices are interconnected through MEC networking and linked to the remote cloud depending on application needs. As the traditional cloud-centered IoT architecture, MEC-IoT architecture is also confronted with security problems. Meanwhile, access authentication is envisioned as the primary problem to be solved. As a physical layer security technology, RFFID is regarded as a lightweight access authentication method, and applicable to the MEC-IoT architecture.

### 2.2. Radio Frequency Fingerprinting Identification (RFFID)

The RFFID method refers to identification based on the radio frequency signal fingerprint of the wireless devices to confirm the access of the legal wireless devices, thereby realizing the identity authentication of the wireless devices. The RFFID, which embodies the hardware property of the wireless transmitters, is difficult to be cloned and can be used for non-cryptographic authentication for the wireless transmitters. Cobb et al. [30] made a further explanation of the mechanism of RFF and introduced electronic component tolerances due to differences in hardware devices, such as printed circuit board traces, integrated circuit internal components, and RF front-end circuits. The electronic component tolerance effect of wireless transmitters is the main reason for generating RFF. Since the hardware of any two wireless devices is different and hard to be faked, it is feasible to uniquely identify electronic components by RF signal fingerprinting.

**Figure 2:**
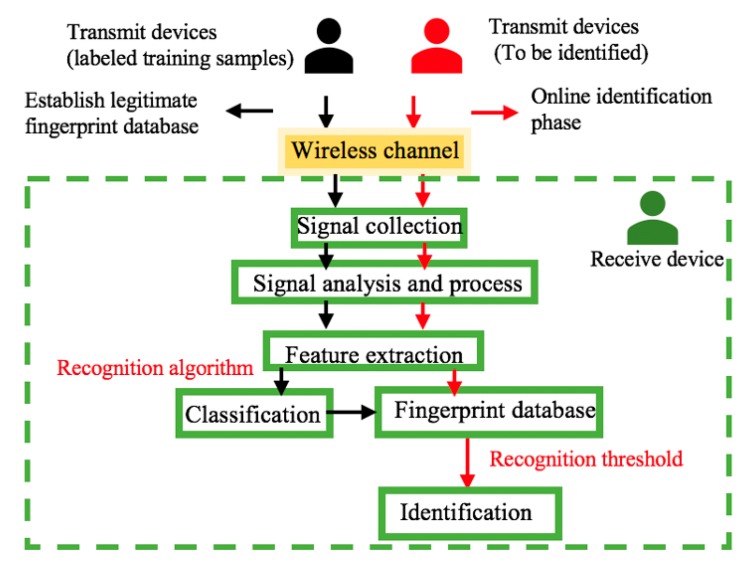
Radio frequency fingerprinting identification (RFFID) authentication method.

As shown in Figure 2, the RFFID method consists of six steps: Signal collection, signal analysis and process, feature extraction and classification, fingerprint database, and identification. RFFID mainly includes two processes. The first one is offline to establish a fingerprint database for legitimate wireless devices by implementing, analyzing and processing the radiation signals after collecting the signals of legitimate devices. The second process is an online authentication process. The signals of the wireless devices to be identified are collected and the fingerprint features are extracted through signal analysis and processing. Then, matching and recognition are carried out in the existing legitimate fingerprint database.

Recently, in order to further improve the authentication rate of RFFID, machine learning method is taken as a recognition algorithm [27,28,29]. However, this method consumes resources in offline samples learning due to a large number of samples training required to ensure the authentication effect. Otherwise, the authentication rate will be compromised if only limited training samples are used. Therefore, abundant computing resources are necessary to guarantee the authentication rate of RFFID. When the computing power of MEC devices is inadequate, it can upload tasks to the cloud platform. By this means, MEC can rationally get computational resources to support and fully ensure the accuracy of tasks. Therefore, a combination of the RFFID method, MEC and cloud can strengthen hardware resource guarantee.

## 3. Security Access Authentication Method Based on RFFID-MEC

**Figure 3:**
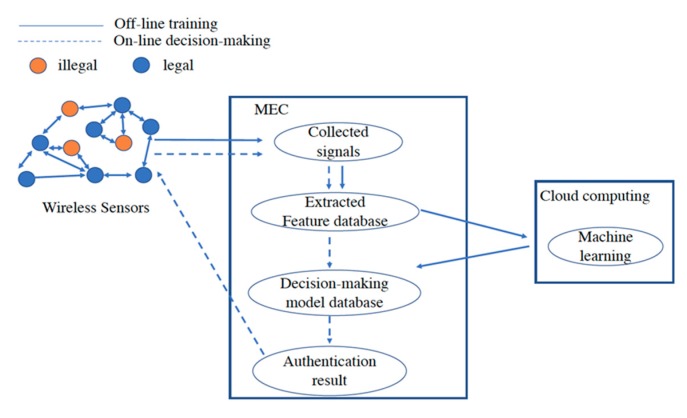
Detailed authentication process.

In this section, we propose a lightweight algorithm for resource-constrained terminals to accomplish access authentication with a satisfied authentication rate. This algorithm that combines the mobile edge computing with the cloud may improve the accuracy of the authentication-based RFFID. The architecture of the proposed RFFID-MEC authentication consists of two layers: The first layer provides signal collection, signal analysis, and process, feature extraction and classification, and establish fingerprint feature database, which will be performed on the MEC layer. The second layer includes learning features, generating decision models, and implementing machine learning algorithms for recognition, which need the powerful computing support for much more complex and resource-consuming tasks, will be implemented on the remote cloud due to the limited computing resources of MEC. Figure 3 shows a detailed logical flow of this authentication process. The authentication process includes two processes that are an offline training process and an online decision-making process.

In the offline training authentication process, a terminal initiates an access request to the MEC platform. After that, the MEC platform collects the signal of the terminal with the identity information, and then performs feature extraction and establishes dynamic fingerprint feature database. The feature information is transmitted to the cloud computing platform. The cloud computing platform makes use of the machine learning algorithm to generate the authentication decision-making model, and transmits the resulted decision model that meets the target authentication rate back to the MEC platform. At this time, the offline training authentication process ends. In the online decision-making authentication process, a terminal initiates an access request to the MEC platform, and the MEC platform collects signals, extracts features, and performs fast identity authentication through the trained authentication model that was established from the previous step.

**Figure 4:**
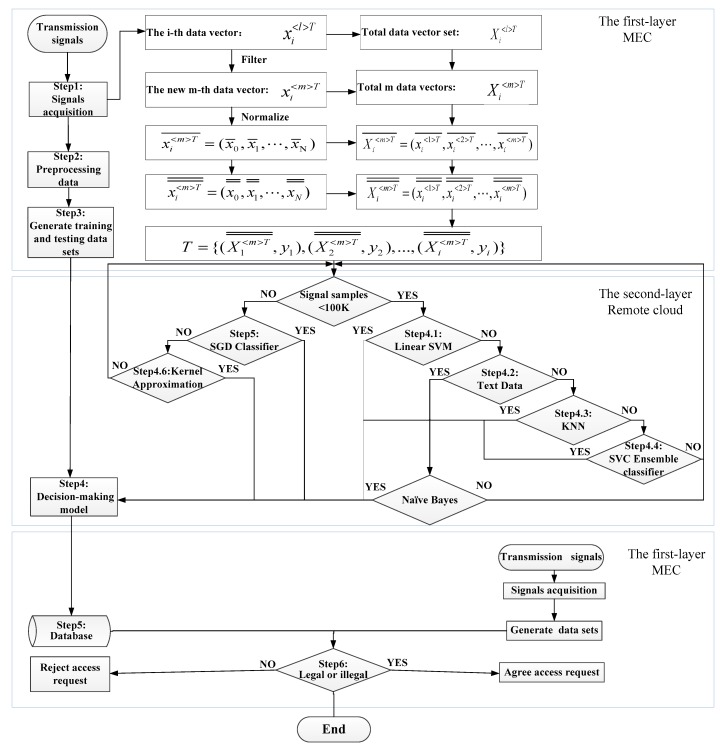
Flow chart of RFFID-MEC method algorithm.

The RFFID-MEC algorithm is illustrated in Figure 4. Notations of frequently-used variables are described in Table 1 for steps. Steps 1–3, 5, 6 are carried out by the first layer, meanwhile Step 4, including Steps 4.1–4.6, are carried out by the second layer.

Step 1: The MEC platform continuously acquires signals:

The MEC platform collects the RF signals of the IoT devices with identity tags:

1. The vector of the l−th collection of the i−th terminal device is: $x_{i}^{<l>T}=(x_{0},x_{1},\ldots ,x_{N})$, (N represents the discrete sample points of the collected signals)
2. The data set of the total L acquisitions of the l−th terminal devices is: $x_{i}^{<l>T}=(x_{i}^{<1>T},x_{i}^{<2>T},\ldots ,x_{i}^{<L>T})$, l=(1,2,⋯,L)

Step 2: Data preprocessing in MEC platform:

The MEC platform preprocesses data sets for filtering and normalizing.

1. According to the data set, we obtain the mean $E(X_{i}^{<l>T})$ and, standard deviation $\sigma_{X^{<l>T}}$, and remove the outliers from the data set $X_{i}^{<l>T}$. Then $x_{i}^{<l>T}$ and, $X_{i}^{<l>T}$ were changed to: $x_{i}^{<m>T}=(x_{0},x_{1},\cdots ,x_{N})$ and, $X_{i}^{<m>T}=(x_{i}^{<1>T},x_{i}^{<2>T},\cdots ,x_{i}^{<m>T})$, m=(1,2,⋯,M), M<L.
2. $x_{i}^{<m>T}=(x_{0},x_{1},\cdots ,x_{N})$ was normalized to new value:
  (1) $$\bar{x}=\frac{1}{N}\sum_{i=1}^{N}x_{i},i=1,2,3,\cdots ,N$$
  (2) $$\sigma^{2}=\frac{1}{N-1}{\sum_{i=1}^{N}\left(x_{i}-\bar{x}\right)}^{2}$$
  (3) $$\bar{x_{i}}=\frac{x_{i}-\bar{x}}{\sigma }$$
  $x_{i}^{<m>T}$ and, $X_{i}^{<m>T}$ were changed to:
  $$\bar{x_{i}^{<m>T}}=(\bar{x_{0}},\bar{x_{1}},\cdots ,\bar{x_{N}})$$
  and
  $$\bar{X_{i}^{<m>T}}=(\bar{x_{i}^{<1>T}},\bar{x_{i}^{<2>T}},\ldots ,\bar{x_{i}^{<m>T}})$$
  where $\bar{x_{i}^{<m>T}}$ has a standard normal distribution with mean zero and unit variance.

Step 3: The MEC platform generates training and testing data sets:

The normalized data sets $\bar{X_{i}^{<m>T}}$ are used by MEC platform to generate the feature vector as the training and testing data sets T as follows:

$\bar{x_{i}^{<m>T}}$ are changed to $\bar{\bar{x_{i}^{<m>T}}}$

(4) $$\begin{array}{c} \varphi_{ik(n)}=f_{i}(n-2^{i+1}k),(i=0,1,\cdots ,J-2)\\ \bar{x_{i}^{<m>T}}=\sum_{i}\sum_{k}\bar{\bar{x_{i}^{<m>T}}}\varphi_{ik}(n) \end{array}$$

$\bar{X_{i}^{<m>T}}$ are changed to $\bar{\bar{X_{i}^{<m>T}}}$.

$$\bar{\bar{x_{i}^{<m>T}}}=(\bar{\bar{x_{0}}},\bar{\bar{x_{1}}},\cdots ,\bar{\bar{x_{{}_{N}}}})$$

$$\bar{\bar{X_{i}^{<m>T}}}=(\bar{\bar{x_{i}^{<1>T}}},\bar{\bar{x_{i}^{<2>T}}},\cdots ,\bar{\bar{x_{i}^{<m>T}}})$$

T is the final generated training data sets given by:

$$T=\{(\bar{\bar{X_{i}^{<m>T}}},y_{1}),(\bar{\bar{X_{2}^{<m>T}}},y_{2}),\ldots ,(\bar{\bar{X_{i}^{<m>T}}},y_{i})\}$$

$$m=(1,2,\ldots ,M),y_{i}\in Y=\{+1,-1\}$$

(+1 is represented as a legal terminal device, −1 is an illegal terminal device.)

Step 4: The cloud platform generates a decision-making model.

Step 4.1: When the number of sample data <100 K, we will choose a support vector machine (SVM) classification algorithm to generate a decision-making model:

(5) $$\begin{array}{l} \min\frac{1}{2}\sum_{i=1}^{N}\sum_{j=1}^{N}\alpha_{i}\alpha_{j}y_{i}y_{j}K(x_{i},x_{j})-\sum_{i=1}^{N}\alpha_{i}\\ s.t\sum_{i=1}^{N}\alpha_{i}y_{i}=0,C\geq \alpha_{i}\geq 0,i=1,2,\ldots ,N \end{array}$$

The decision-making model using the linear kernel function $K(\vec{x},\vec{z})=\vec{x}\cdot \vec{z}$, is applied to the linear classification of large data sets to find the optimal solution:${\vec{\alpha }}^{*}={\left(\alpha_{1}^{*},\alpha_{2}^{*},\cdots ,\alpha_{N}^{*}\right)}^{T}$, ${\vec{w}}^{*}=\sum_{i=1}^{N}\alpha_{i}^{*}y_{i}\vec{x_{i}}$, choosing $C>\alpha_{j}^{*}>0$, $b^{*}=y_{j}-\sum_{i=1}^{N}\alpha_{i}^{*}y_{i}K(\bar{\bar{X_{i}^{<m>T}}},\bar{\bar{X_{j}^{<m>T}}})$.

The decision-making model is defined by:

(6) $$f(\vec{x})=sign(\sum_{i=1}^{N}\alpha_{i}^{*}y_{i}K(\bar{\bar{X_{i}^{<m>T}}},\bar{\bar{X_{j}^{<m>T}}})+b^{*})$$

According to the training data sets testing model, if it can satisfy the correct target recognition rate, the current model is the decision-making model and transmitted to the MEC platform database, otherwise the algorithm will jump into Step 4.2.

Step 4.2: The cloud platform determines whether the data sets are text data. (a) If they are text data, using Naive Bayes, which can achieve the correct target recognition rate, then the current model is the decision-making model and transmitted to the MEC platform database, otherwise the algorithm will jump to Step 4. (b) If it is not text data, the algorithm will jump to Step 4.2.

Step 4.3: The cloud platform uses the (k-nearest neighbor) KNN classification algorithm to determine whether the correct recognition rate is greater than the preset one.

(a) Input the training data set T:
  $$T=\{(\bar{\bar{X_{1}^{<m>T}}},y_{1}),(\bar{\bar{X_{2}^{<m>T}}},y_{2}),\cdots ,(\bar{\bar{X_{i}^{<m>T}}},y_{i})\}$$
  $\bar{\bar{X_{i}^{<l>T}}}\in \chi \subset R^{n}$ is the feature of the instance, and $y_{i}\in Y=\{+1-1\}$ is the category of the instance.
(b) Calculate the Euclidean distance:
  (7) $$L_{p}(\bar{\bar{X_{i}^{<m>T}}},\bar{\bar{X_{j}^{<m>T}}})={\left({\sum_{m=1}^{M}\left|\bar{\bar{x_{i}^{<m>T}}}-\bar{\bar{x_{j}^{<m>T}}}\right|}^{2}\right)}^{\frac{1}{2}}$$
(c) Find the k samples closest to $\bar{\bar{x_{i}^{<m>T}}}$ in the training data sets T, let the neighborhood of this t point be $N_{t}(\bar{\bar{x_{i}^{<m>T}}})$.
(d) Determine the category of $\bar{\bar{x_{i}^{<m>T}}}$ in $N_{t}(\bar{\bar{x_{i}^{<m>T}}})$, according to the classification decision is $y_{i}$:
  (8) $$\begin{array}{l} y=\underset{C_{j}}{\arg\max}\sum_{\bar{\bar{x_{i}^{<m>T}}}\in N}I(y_{s}=c_{j})\\ \left(s=1,2,\cdots ,M*i;j=1,2\right) \end{array}$$
  where I is the indicator function. If $y_{s}=c_{j}$, then $I\left(y_{s}=c_{j}\right)$ is 1. By a similar argument, if $y_{s}\neq c_{j}$, then $\;I\left(y_{s}=c_{j}\right)$ is 0.

According to the training data sets testing model, if it can satisfy the correct recognition of the target, the current model is the decision model and transmitted to the MEC platform database, otherwise the algorithm will jump to Step 4.4.

Step 4.4: The cloud platform uses the integrated classifier to determine whether the correct recognition rate is greater than the preset. Integrated classifier, using a variety of existing learning algorithms from the training data to generate individual learners and based on Adaboost binary classification algorithm process, is as follows:

(a) Input the training data set T:
  $$T=\{(\bar{\bar{X_{1}^{<m>T}}},y_{1}),(\bar{\bar{X_{2}^{<m>T}}},y_{2}),\cdots ,(\bar{\bar{X_{i}^{<m>T}}},y_{i})\}$$
(b) Initialize the weight distribution of training data
  $$D_{1}=(w_{11}w_{12}\cdots w_{1q}\cdots ,w_{1i}),w_{1q}=\frac{1}{i}q=1,2,\cdots ,i$$
(c) Use the $D_{h}(h=1,2,\cdots ,H)$ training data set with weights to learn to get the basic classifier
  $$G_{h}(\bar{\bar{x_{i}^{<m>T}}}):X\to \{-1,+1\}$$
  Calculate the classification error rate of $G_{h}(\bar{\bar{x_{i}^{<m>T}}})$ on the training data set given by:
  $$\begin{array}{ll} e_{h} & =P(G_{h}(\bar{\bar{x_{i}^{<m>T}}})\neq y_{i})\\ & =\sum_{q=1}^{i}w_{hq}I(G_{h}(\bar{\bar{x_{i}^{<m>T}}})\neq y_{i}) \end{array}$$
  Calculate the coefficients of $G_{h}(\bar{\bar{x_{i}^{<m>T}}})$
  (9) $$\alpha_{h}=\frac{1}{2}\log\frac{1-e_{h}}{e_{h}}$$
  Update the weight distribution of the training data sets:
  (10) $$\begin{array}{l} D_{h+1}=(w_{h+1,1}\cdots w_{h+1,q}\cdots ,w_{m+1,i})\\ w_{h+1,q}=\frac{w_{hq}}{z_{h}}\exp(-\alpha_{h}y_{q}G_{h}(\bar{\bar{x_{i}^{<m>T}}}))\\ \left(q=1,2,\cdots ,i\right) \end{array}$$
  $z_{h}$ is a normalization factor:
  (11) $$z_{h}=\sum_{q=1}^{i}w_{hq}\exp(-\alpha_{h}y_{q}G_{h}(\bar{\bar{x_{i}^{<m>T}}}))$$
  It makes $D_{h+1}$ a probability distribution.
(d) Build a linear combination of basic classifiers:
  (12) $$f(x)=\sum_{h=1}^{H}\alpha_{h}G_{h}(\bar{\bar{x^{<m>T}}})$$
(e) Get the final classifier:
  (13) $$\begin{array}{ll} G(\bar{\bar{x^{<m>T}}}) & =\mathrm{si}gn(f(x))\\ & =\mathrm{si}gn(\sum_{h=1}^{H}\alpha_{h}G_{h}(\bar{\bar{x^{<m>T}}})) \end{array}$$

According to the testing set of test models, if it can satisfy the correct target recognition rate, the current model is the decision-making model and transmitted to output the MEC platform database, otherwise the algorithm will jump to Step 4.

Step 4.5: When the number of sample data is greater than 100 K, the SVM classification algorithm based on stochastic gradient descent is selected and the cost model is optimized by stochastic gradient descent method given by:

(14) $$\begin{array}{ll} J(\theta ) & =\frac{1}{s}\sum_{i=1}^{s}\frac{1}{2}{\left(y^{i}-h_{\theta }(x^{i})\right)}^{2}\\ & =\frac{1}{s}\sum_{i=1}^{s}\cos t(\theta ,(x^{i},y^{i})) \end{array}$$

The final decision-making model is an SVM algorithm based on the multi-class linear kernel. According to the testing data sets testing model, if it satisfies the correct target recognition rate, the current model is a decision-making model and transmitted to the MEC platform database, otherwise the algorithm will jump to Step 4.6 and continue to select the classification algorithm.

Step 4.6: Kernel approximation is a nonlinear classification model. Nonlinear SVM is a classification model based on linear SVM. Different kernel functions are used to realize the transformation of high-dimensional space map to low-dimensional space. The optional kernel functions are:

(15) $$\begin{array}{l} k(\vec{x},\vec{z})={\left(\gamma (\vec{x}\vec{z}+1)+r\right)}^{p}\\ k(\vec{x},\vec{z})=\exp(-\gamma {\left\|\vec{x}-\vec{z}\right\|}^{2})\\ k(\vec{x},\vec{z})=\tanh(\gamma (\vec{x}\vec{z})+r)\\ k(x,y)=\sum_{i}\frac{2x_{i}y_{i}}{x_{i}+y_{i}}\\ k(x,y)=\prod_{i}\frac{2\sqrt{x_{i}+c}\sqrt{y_{i}+c}}{x_{i}+y_{i}+2c} \end{array}$$

According to the testing set test model, if it satisfies the correct target recognition rate, the current model is the decision-making model and transmitted to the MEC platform database, otherwise the algorithm will jump to Step 4.

Step 5: The MEC platform stores the decision-making model and data set in the database.

Step 6: The MEC platform implements access authentication to determine whether it is legal.

Step 7: The MEC platform continuously collects the RF signals of the IoT devices with identity tags: The MEC platform collects the signal and preprocesses the data, and then passes the processed data and the training data set in the database through the decision model to judge whether the terminal identity is legal. If it is the legal device, MEC platform consents the access request. If it is not legal, the MEC platform refuses to access the request.

The main features of the RFFID-MEC architecture are:

1. Low-complexity: There is no need for encryption algorithm at the terminal node, and all the identification algorithms are completed by MEC. Therefore, the novel authentication method is especially beneficial to the terminals that are resource-constrained.
2. Low-latency: As the decision-making model has been generated by cloud computing and transmitted to MEC platform, it considerably reduces decision latency. This becomes particularly important for IoT scenarios, for example, when dealing with a large number of legitimate users’ access requests that need low latency and real-time access authentication such as a driverless scenario.
3. Universality: This method is suitable for interconnection of resource-constrained IoT devices in 5G networks. Meanwhile, it has the characteristics of low computational complexity and high authentication accuracy.

## 4. RFFID-MEC Authentication Method Evaluation

**Figure 5:**
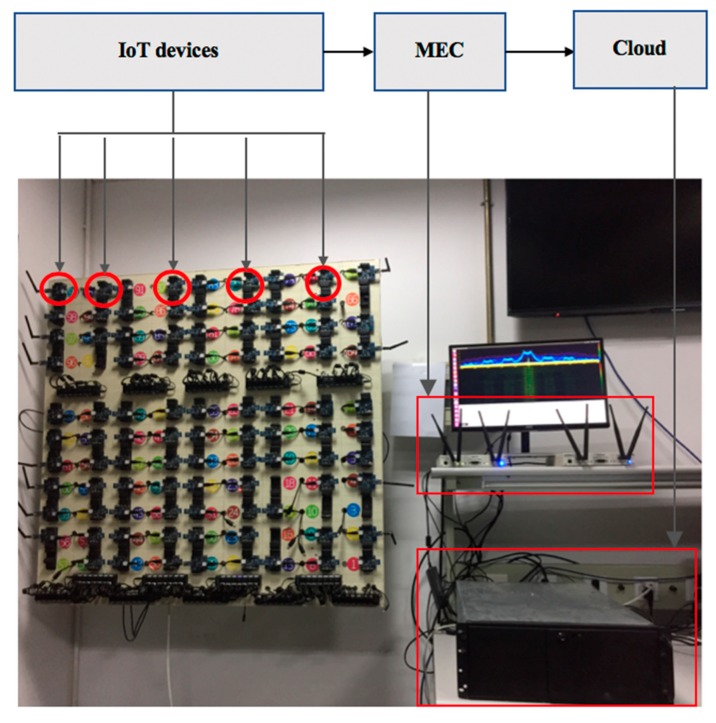
Typical application scenarios of RFFID-MEC authentication method.

We demonstrate a typical application scenario of RFFID-MEC Authentication method, as illustrated in Figure 5. IoT terminals are many NRF24Les nodes. More specifically, NRF24LE is a single RF transceiver chip and the operating frequency range is from 2.4 to 2.525 GHz. Its internal components include frequency synthesizer, power amplifier, crystal oscillator, GFSK modulator, and filters. NRF24LE chip is characterized by small power consumption, monolithic and small size. It is widely used in home automation and factory control [34]. MEC platform was composed of Universal Radio Software Peripheral (USRP). USRP is an open-source software-defined radio platform, which is consisted of a mother-board equipped with a dual 14-bit analog to digital converter (ADC) operating at 100 MHz and dual 16-dit digital to analog converter (DAC) operating at 400 MHz, and two UBX160 daughter boards and vert2450 antennas. UBX160 transceiver daughter boards that act as a front end and have a frequency range from 10 MHz to 6 GHz, which allows transmitting and receiving in the 2.4 GHz industrial, scientific, and medical radio band (ISM band) [35]. Cloud server is taken as a cloud platform.

There are several steps in our experiment:

The steps of the offline training authentication are as follows.

Step 1: Two terminal nodes with identity tags (illegal, legal) send signals to MEC platform, then the MEC platform collects the signals;

Step 2: The MEC platform preprocesses the collected signals to establish a fingerprint feature database;

Step 3: The MEC platform generates training and testing data sets, which are transmitted to the cloud platform;

Step 4: The cloud platform performs the training processing and generates a decision-making model, which is transmitted back to the MEC platform;

Step 5: The MEC platform stores the decision-making model. Online decision-making authentication includes one step as follows:

Step 6: Terminals (illegal, legal) send signals to the MEC platform. The MEC platform generates feature data sets and determines whether it is legal or not via a previously trained model.

**Figure 6:**
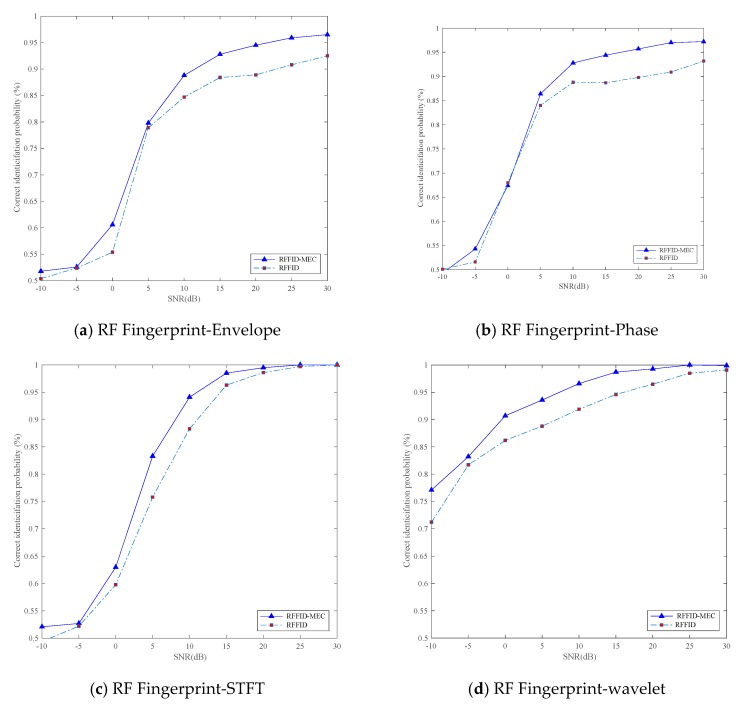
Correct identification probability versus SNR for RFFID-MEC and RFFID using four different RF fingerprint features including: Envelope, phase, STFT, and wavelet feature.

In addition, we have compared RFFID-MEC with traditional RFFID methods. Our simulation utilizes each of four kinds of RF fingerprint features to verify the authentication effect under different SNR. Compared with the traditional RFFID method, the RFFID-MEC method takes advantage of the cloud computing platform to increase the number of offline training samples in a machine learning algorithm. As shown in Figure 6, from the four simulation results, it can be seen that whether using envelope, phase, STFT, or wavelet feature, the correct identification probability of RFFID-MEC method is higher than that of RFFID method at different SNR. Besides, the simulation indicates that the correct identification probability of wavelet feature clearly outperforms the other ones and achieves a higher correct identification rate at low SNR, because the fingerprint of wavelet transform possesses strong anti-noise characteristics [36]. Therefore, we demonstrate the effectiveness of the proposed RFFID-MEC method choosing wavelet RF feature, which can be applied to security authentication.

## 5. Conclusions

The paper has developed a lightweight RFFID-MEC authentication method by taking advantage of attributes-based MEC, cloud, and non-encryption RFFID for IoT terminals. The presented two-layer model is extremely suitable for the MEC-based IoT paradigms. Compared with the traditional RFFID security access authentication, our light-weight RFFID-MEC authentication method has achieved higher authentication accuracy and improved the work efficiency of IoT terminals, in which all the computing burdens are taken by the edge devices and the cloud. Subsequently, we put this method into an application scenario. Our simulations have demonstrated the effectiveness of this method in actual IoT environments.

## Figures and Tables

**Table 1:** Notations of frequently-used variables.

Symbol	Description
i	The i-th terminal
N	Discrete points of signal acquisition
$x_{i}^{<l>T}$	The l-th collection of the i-th terminal’s vector
$X_{i}^{<l>T}$	The total l-th times collection of the i-th terminal’s set
$x_{i}^{<m>T}$	The vector after remove the outline from the $x_{i}^{<l>T}$
$X_{i}^{<m>T}$	The set after remove the outline from the set $X_{i}^{<l>T}$
$\bar{x_{i}^{<m>T}}$	The data normalization of vector $x_{i}^{<m>T}$
$\bar{X_{i}^{<m>T}}$	The data normalization of set $X_{i}^{<m>T}$
$\bar{\bar{x_{i}^{<m>T}}}$	The vector $\bar{x_{i}^{<m>T}}$ generated after DTWT
$\bar{\bar{X_{i}^{<m>T}}}$	The set $\bar{X_{i}^{<m>T}}$ generated after DTWT
T	The training data set
$y_{i}$	The category of the instance
